# A combination of machine learning and infrequent metadynamics to efficiently predict kinetic rates, transition states, and molecular determinants of drug dissociation from G protein-coupled receptors

**DOI:** 10.1063/5.0019100

**Published:** 2020-09-23

**Authors:** João Marcelo Lamim Ribeiro, Davide Provasi, Marta Filizola

**Affiliations:** Department of Pharmacological Sciences, Icahn School of Medicine at Mount Sinai, New York, New York 10029, USA

## Abstract

Determining the drug-target residence time (RT) is of major interest in drug discovery given that this kinetic parameter often represents a better indicator of *in vivo* drug efficacy than binding affinity. However, obtaining drug-target unbinding rates poses significant challenges, both computationally and experimentally. This is particularly palpable for complex systems like G Protein-Coupled Receptors (GPCRs) whose ligand unbinding typically requires very long timescales oftentimes inaccessible by standard molecular dynamics simulations. Enhanced sampling methods offer a useful alternative, and their efficiency can be further improved by using machine learning tools to identify optimal reaction coordinates. Here, we test the combination of two machine learning techniques, automatic mutual information noise omission and reweighted autoencoded variational Bayes for enhanced sampling, with infrequent metadynamics to efficiently study the unbinding kinetics of two classical drugs with different RTs in a prototypic GPCR, the μ-opioid receptor. Dissociation rates derived from these computations are within one order of magnitude from experimental values. We also use the simulation data to uncover the dissociation mechanisms of these drugs, shedding light on the structures of rate-limiting transition states, which, alongside metastable poses, are difficult to obtain experimentally but important to visualize when designing drugs with a desired kinetic profile.

## I. INTRODUCTION

A large number of clinically used drugs elicit their biological effects via G protein-coupled receptors (GPCRs).^1^ Despite decades of drug discovery efforts focused on this important family of membrane proteins, many drug candidates eventually fail in clinical trials because of their lack of *in vivo* efficacy or safety. For years, researchers have focused on developing potent and selective GPCR ligands using their binding affinity, i.e., the strength of association of a drug to its receptor at equilibrium, as a surrogate for *in vivo* efficacy. Unfortunately, affinity quantifications such as the dissociation equilibrium constant, K_d_, can be unreliable predictors of a drug’s *in vivo* efficacy, most likely due to variable concentrations of free drug in a living organism driving the system away from equilibrium. Kinetic quantities such as the rates at which a drug associates with its target (k_on_), or dissociates from (k_off_), have been suggested to play a role that is as important as, and possibly even more important than, the binding affinity in determining the *in vivo* efficacy of a drug. For instance, a retrospective assessment^2^ of 50 marketed drugs on 12 different targets revealed that about 70% of these drugs exhibited a long drug-target residence time (RT), which is defined as the lifetime of the drug-target complex and is equal to the inverse of k_off_. These slow dissociating drugs displayed higher therapeutic effects in the clinic when compared to faster dissociating drugs, whereas the latter exhibited reduced on-target side effects and were in general less harmful.

Based on these considerations, it has become clear in the drug discovery field that kinetic properties of drug-target complexes, and in particular dissociation rate constants or RTs, may represent better surrogate markers of the clinical efficacy of drugs,^3,4^ offering an important criterion for selecting candidate molecules for clinical development. However, obtaining accurate dissociation rate constants either experimentally or computationally is far from trivial. This is particularly true for GPCRs whose complex physiological context and limited access to chemical and biophysical tools present additional challenges in accurately measuring binding kinetics.^5^ For computational methods such as molecular dynamics (MD) simulations, the main challenge in studying GPCR binding kinetics is that the timescale for drug dissociation from a GPCR is often on the order of minutes or longer, and therefore inaccessible with standard simulations. Recently, however, computational methods using either powerful frameworks for generating and analyzing MD data or enhanced sampling algorithms have begun to be investigated in their ability to efficiently predict accurate k_off_ values,^6^ including in applications to GPCRs. In addition to providing k_off_ estimates, these approaches can unveil a plethora of experimentally inaccessible information, including atomic-resolution structures of transition states (TS) and metastable states involved in binding, which are hard to determine experimentally due to their transient nature. Yet, this information is very important for the purpose of drug discovery as it informs ways to alter a drug’s kinetic profile and with that its efficacy *in vivo*.

One computational approach that has been successfully used to extract kinetic properties of drug–protein (un)binding involves building a Markov State Model (MSM) from data obtained from a large number of short MD simulations carried out with or without adaptive sampling protocols (e.g., see Refs. 7–9). These simulations typically require several million CPU or GPU hours on fairly expensive special-purpose computing resources, which are not accessible to many investigators.^10,11^ To overcome these limitations, a number of enhanced sampling methods^12^ have been proposed to reduce the time required to observe drug dissociation. One such method, which is capable of recovering unbiased kinetics from metadynamics simulations, is infrequent metadynamics.^13^ One of the crucial assumptions of this method is that the transition state remains bias-free during the course of the biased simulations, a condition that can be satisfied if the bias deposition is infrequent. Although infrequent metadynamics has now been applied to several systems,^14–20^ including GPCRs,^15^ identifying an appropriate reaction coordinate (RC) to describe the biological process of interest continues to pose a considerable challenge to the successful application of this protocol to complex systems.

In the work that follows, we validate a computational protocol that combines machine learning and infrequent metadynamics to efficiently study the typical unbinding of classical GPCR small-molecule drugs in the minute regime, using the μ-opioid receptor (MOPr) as a prototypic GPCR system. Specifically, we use the Automatic Mutual Information Noise Omission (AMINO) method^21^ to screen through a large number of molecular features and select a subset of them that can then be used in the Reweighted Autoencoded Variational Bayes for Enhanced sampling (RAVE) method^22,23^ to learn optimal reaction coordinates for infrequent metadynamics. The purpose of applying AMINO before RAVE is that it enables the selection of non-redundant molecular features in a robust and automated manner instead of manually selecting them through visual inspection. We show that the strategy is able to efficiently sample the dissociation of two classical opioid receptor drugs with significantly different k_off_ values (1.388 ± 0.1 min^−1^ for morphine and 0.106 ± 0.02 min^−1^ for buprenorphine^24^) and to provide estimates of dissociation rates within an order of magnitude from experimental values. A nudged elastic band (NEB) algorithm applied to an analytical Gaussian Mixture Model (GMM) energy landscape^25^ generated from the aforementioned metadynamics simulations provided mechanistic insights into the explored unbinding pathways of morphine and buprenorphine, as well as information about the various metastable states and transition states that may or may not act as kinetic traps along these pathways. Although it does not offer a high-throughput strategy for the determination of drug residence times, the proposed combination of the aforementioned tools with infrequent metadynamics represents a step forward in enhancing existing rational drug design approaches for GPCRs. In particular, the strategy contributes (a) estimates of kinetic rates at a much reduced computational cost (drug dissociation requiring 1 min–10 min are achieved in microsecond-timescale simulations, corresponding to ∼7 orders of magnitude speed-up), (b) atomic-resolution structures of metastable states and transition states along the drug unbinding pathway, which are difficult or impossible to determine experimentally, and (c) molecular determinants of drug-receptor binding kinetics whose modulation may guide the design of improved therapeutics.

## II. COMPUTATIONAL DETAILS

**FIG. 1.**
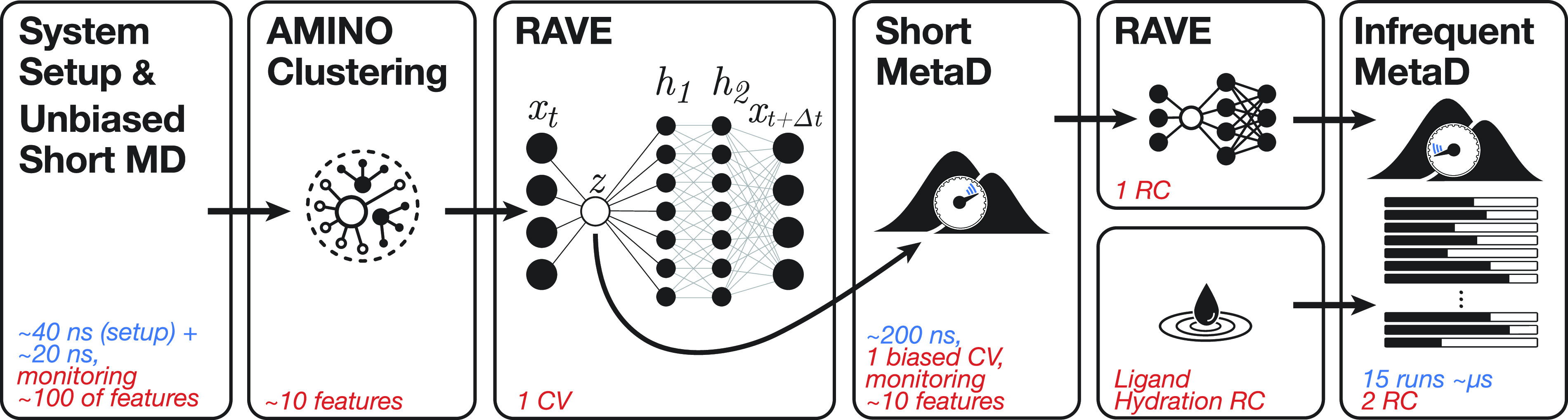
Overall schematic of the computation protocol. The first panel indicates that the protocol starts with the system setup followed by a short unbiased MD simulation in which we track the time evolution of 145 molecular features for about 20 ns. The second panel represents the use of AMINO to cluster these molecular features into 10–20 representatives that best describe ligand unbinding from the receptor. The third panel indicates that RAVE is used to learn a first 1D collective variable (CV) or trial reaction coordinate (RC) from the AMINO-derived features. The fourth panel indicates that a metadynamics simulation is run with bias deposited along the first RAVE-based CV while tracking the time evolution of the AMINO-derived features. In the top half of the fifth panel, the final RAVE-based RC is generated using the metadynamics data collected during training, while in the bottom half the ligand’s hydration state is defined as another RC. The final panel indicates the production-grade infrequent metadynamics simulations that were run by biasing the potential along these two RCs.

An overall schematic of the computation protocol is provided in Fig. 1, and additional details of the computations are provided in Table S1.

### A. Setup and MD simulations of the ligand–receptor complex systems

Morphine and buprenorphine were docked into the crystal structure of the active MOPr (5C1M)^26^ by aligning the heavy atoms of their alkaloid scaffolds to corresponding ones in the co-crystal ligand BU72. N- and C-terminal residues (S64 and I352) of the receptor were capped with an N-terminal acetyl and a C-terminal N-methyl amide, respectively, using Maestro. Furthermore, the missing residues in helix 8 (R348, E349, F350, C351, and I352) of the active mouse MOPr were modeled using the coordinates of the MOPr inactive crystal structure (4DKL).^27^ An 80 × 80 Å^2^ 1-palmitoyl-2-oleoyl-sn-*glycero*-3-phosphocholine (POPC)/10% cholesterol bilayer with TIP3P water molecules was generated using the CHARMM-GUI webserver^28^ and equilibrated by MD without restraints for 20 ns. The ligand-bound MOPr complex was then placed in this membrane using the *inflategro* script^29^ and a deflation ratio of 0.97. The system was neutralized with a 0.15M concentration of NaCl. Force field parameters for morphine and buprenorphine were obtained from the CHARMM General Force Field ParamChem webserver,^30^ and optimized and verified following the established protocols.^30^ Ligand-bound MOPr systems were energy minimized and then equilibrated by (a) running a 1-ns MD simulation in the NVT ensemble with restraints on the ligand, the receptor, and lipids, (b) relaxing these restraints over 20 ns of MD simulation in the NPT ensemble, and (c) carrying out a final 40-ns MD simulation without restraints. The quality of the system setup was assessed by monitoring several properties of the protein and the membrane patch during the unrestrained equilibration runs. These included the total area of the lipid membrane [including the space occupied by the receptor transmembrane (TM) region] as well as the lipid temperature, and the corresponding plots are shown in Fig. 1 of the supplementary material for the buprenorphine-MOPr system as an example.

### B. Learning optimal reaction coordinates to simulate drug-receptor dissociation

In order to learn optimal reaction coordinates for achieving drug dissociation from a prototypic GPCR while appropriately describing the slow dynamics of the system, we used two recently published machine learning methods. The first of these methods, called AMINO,^21^ allowed us to extract non-redundant molecular features for describing the drug-GPCR (un)binding process from a large number of initial features that are clustered based on their mutual information. Specifically, an initial set of 145 molecular features defined as the distances between the center of mass (COM) of morphine or buprenorphine relative to every other MOPr residue were input into AMINO as a time series generated from a 20-ns unbiased MD simulation with a 1-ps time resolution. The resulting dataset of 20 000 × 145 features was analyzed by AMINO using default settings to produce up to 20 clusters. The representative features of these clusters were then used as an input to learn an optimal reaction coordinate using a second machine learning method called RAVE.^22,31^ Specifically, using a representative molecular feature from each AMINO-based cluster as an input, RAVE employs the past–future information bottleneck framework^32–34^ to produce a low-dimensional (bottleneck or latent space) representation of the data, which has maximal predictive power of the time evolution of molecular features obtained from MD simulations, and can serve as an affordable proxy for an optimal reaction coordinate.

Specifically, the following neural network (NN) architecture was used to learn the reaction coordinate for simulating morphine or buprenorphine dissociation from the MOPr. First, a simple encoder was used to linearly map the *k* AMINO-identified molecular features (where *k* = 9 or *k* = 20 for morphine or buprenorphine simulations, respectively) into a 1D bottleneck via a single inner product, *z* = ***w*** *·* ***x***, where ***x*** is the *k*-dimensional input feature vector, ***w*** is the *k*-dimensional vector of NN coefficients, and *z* is the scalar bottleneck value. Meanwhile, the decoder was defined as two sequential non-linear transformation layers with dimension *K* = 128, followed by a linear transformation. The non-linear transformation to a *K*-dimensional space can be specified by

$$h=f\left(W_{2}f\left(W_{1}z+b_{1}\right)+b_{2}\right).$$ (1)

In Eq. (1), *W*_1_ is the *K* × 1 matrix of coefficients, *b*_1_ is the *K*-dimensional vector of constants, *W*_2_ is the *K* × *K* matrix of coefficients, *b*_2_ is the *K*-dimensional vector of constants, and *f* represents the element-wise non-linearity, specifically, the exponential linear unit function.^35^ The final transformation back to the *k*-dimensional feature space of the data, which does not include any non-linearity, is described as

$$x_{\Delta t}=W_{3}h+b_{3}.$$ (2)

In Eq. (2), *W*_3_ is the *k* × *K* matrix of coefficients and *b*_3_ is the *k*-dimensional bias. The network is trained using the coordinates propagated forward by a small time increment, which we indicate with ***x***_Δ*t*_.

The NN architecture described above was trained using the RMSprop optimizer for 5000 epochs and a learning rate of 0.0002. Specifically, we used the mean square error as the objective function, which was reweighted for trajectories generated herein by metadynamics (see below). The reweighting scheme included the correction introduced in Ref. 23, which accounts for the effect of the bias on the time series through its “corruption” of the propagator. Since the correction holds for short time lags in the data, we set Δ*t* to 2 ps. At convergence, the fractional loss, i.e., the value of the loss relative to the first training epoch, was 0.1 for the autoencoder trained for morphine and 0.05 for buprenorphine.

Since drug dissociation from the MOPr may have timescales longer than minutes, the reaction coordinate obtained from the NN trained on the initial unbiased 20-ns MD simulations is unlikely to provide a thorough description of the drug-receptor dissociation process. Thus, we used the initial reaction coordinate identified by RAVE to run a metadynamics simulation (see details below) aimed at enhancing the exploration of the drug-receptor dissociation pathway. The time series of the molecular features derived from this initial metadynamics simulation was then used to train the NN with a more robust dataset to obtain a better approximation of the reaction coordinate. The infrequent metadynamics simulations used as production runs (see below) were performed biasing this reaction coordinate as well as a collective variable defined by another important contributor of dissociation kinetics, namely, the hydration state of the ligand (e.g., see Ref. 16), as defined in Sec. II C.

### C. Metadynamics simulations

The well-tempered metadynamics simulation carried out to learn a better reaction coordinate with RAVE used an initial hill height of 0.5 kJ/mol, a hill width of 0.05, a bias factor of 20, and a bias deposition interval of 25 ps. The same bias factor, hill height, and hill width were used for production runs consisting of infrequent, well-tempered, metadynamics simulations along a two-dimensional space defined by the aforementioned RAVE reaction coordinate derived from initial well-tempered metadynamics simulation data and the solvation of the ligand, which was defined using the following rational switching function:

$$s\left(r\right)=\frac{1-{\left(\frac{r}{R_{0}}\right)}^{6}}{1-{\left(\frac{r}{R_{0}}\right)}^{10}},$$

where *R*_0_ is 0.3 nm and *r* is the distance between the COM of the ligand and oxygen atom of a water molecule. The collective variable (CV) was defined as the sum $S=\sum_{i}s\left(r_{i}\right)$ over all water molecules in the simulation. For these infrequent metadynamics production runs, three different bias deposition intervals, specifically, 20 ps, 30 ps, and 50 ps, were used to evaluate the speed-accuracy trade-off in simulating drug dissociation from the MOPr, using the morphine-MOPr system as a test case. Based on these results, we ran a single batch of infrequent metadynamics simulations with a bias deposition interval of 30 ps to study the dissociation of buprenorphine from the MOPr. All metadynamics simulations were run using the GROMACS^36^ 2018.6 engine patched with Plumed^37^ (version 2.5.1). All production runs (a total of 15 independent simulations for each MOPr system) used the leap-frog integrator to solve Newton’s equation of motion using a time step of 4 fs and hydrogen mass repartitioning.

### D. Calculation of dissociation rate constants

Kinetic rates were calculated using an established protocol to rescale the biased time in metadynamics simulations back to the unbiased time.^13,38^ This is possible if the transition state region sampled during biased simulations is kept bias-free, which is likely the case if the bias deposition is infrequent.^13^ Specifically, the unbiased dissociation time *t* for each independent infrequent metadynamics run was calculated as

$$\begin{array}{c} t=\underset{t_{i}<t_{D}}{\sum }\Delta \tau e^{\beta V\left(t_{i}\right)} \end{array},$$ (3)

where Δ*τ* is the time step, *β* = (*k*_*b*_*T*)^−1^ with *k*_*b*_ representing the Boltzmann constant and *T* is the temperature, *V*(*t*_*i*_) is the bias experienced at time *t*_*i*_, and the sum is extended until the time *t*_*D*_ in which the ligand dissociates from the receptor. The latter corresponds here to the first time that the ligand’s COM distance from the conserved residue D147^3.32^ exceeds 42.5 Å for morphine and—due to the larger ligand size—55 Å for buprenorphine. Residue numbers here and thereafter refer to the mouse MOPr sequence with most superscript numbers referring to the generic numbering scheme by Ballesteros and Weinstein,^39^ unless structural considerations (e.g., location in loops and helix gaps) required using the numbering scheme by Isberg *et al.*^40^ Thus, in the superscript used throughout the text, the first number corresponds to the transmembrane (TM) helix the residue belongs to, the second number is counted relative to the most conserved residue in that helix, which is set to 50, and the third number (when used) reflects a necessary adjustment based on structural considerations.

The ligand’s residence time, *τ*, which is the inverse of the dissociation rate constant (i.e., *τ* = *k*_off_^−1^), could in principle be obtained by averaging the ligand dissociation times calculated with Eq. (3) over the several infrequent metadynamics simulations carried out on the morphine-MOPr and buprenorphine-MOPr systems. However, ligand unbinding from a receptor is a rare event, and we expect individual dissociation times to be exponentially distributed around *τ*, i.e., *p*(*t*_*i*_ = *t*) ∝ exp(*t*/*τ*), making their average a noisy estimator of *τ*. To estimate *τ* more efficiently, we take a different approach, which shares similarities with the work originally proposed by Salvalaglio *et al.*^41^ In order to incorporate inherent simulation errors (i.e., free energy errors and activation energy errors), we allow for the dissociation times *t*_*i*_ calculated from each individual metadynamics simulation to be exponentially distributed with a simulation-dependent parameter, *τ*_*i*_, which models the error deriving from the individual simulations,

$$\begin{array}{c} p\left(t_{i}=t|\tau_{i}\right)={\tau_{i}}^{-1}e^{t\;/\tau_{i}} \end{array}.$$ (4)

We then model the spread of *τ*_*i*_ around the true residence time *τ* using a log-normal distribution

$$\begin{array}{c} p\left(\text{log}\left(\tau_{i}\right)|\tau ,\sigma \right)={\left(\sigma \sqrt{2\pi }\right)}^{-1}e^{-\frac{1}{2}{\left(\frac{\text{log}\left(\tau_{i}\right)-\text{log}\left(\tau \right)}{\sigma }\right)}^{2}} \end{array}$$ (5)

with standard deviation *σ*. A large *σ* reflects large variations in the inherent dissociation times from the different simulations, while for *σ* → 0 we obtain *τ*_*i*_ → *τ* as proposed by Salvalaglio *et al.*^41^ in the error free regime. Because the main source of error in MD simulations tends to be contained in the underlying free energy surface (FES), we model the prior distribution for *σ* as a log-normal whose center reflects the typical uncertainty in the underlying FES, i.e., Δ*E*/*k*_*b*_*T* ∼ 1, in the form of

$$\begin{array}{c} \text{log}\left(\sigma \right)∼N\left(1,2\right) \end{array}.$$ (6)

Using Bayes’ relation to express the posterior distribution of the dissociation time and the spread *σ* from Eqs. (4)–(6) leads to

$$p\left(\tau ,\tau_{i},\sigma \text{|}t_{i}\right)\propto p\left(t_{i}\text{|}\tau_{i}\right)p\left(\tau_{i}\text{|}\tau ,\sigma \right)p\left(\sigma \right)p\left(\tau \right),$$ (7)

where the posterior $p\left(\tau ,\tau_{i},\sigma \text{|}t_{i}\right)$ here defines the distribution of the residence time *τ* and *p*(*τ*) is a non-informative prior distribution for *τ*. Specifically, we use a Cauchy distribution on *τ*, represented by *λ* = 1/*τ*. Values for *τ* as well as their 75% credible intervals are obtained from sampling this posterior and used as estimates for RT. The same posterior also provides estimates of the spread parameter *σ*. Similar to the work of Salvalaglio *et al.*,^41^ we use the Kolmogorov–Smirnov (KS) test to validate the model.

### E. Ligand unbinding pathways and transition state characterization

Next, we used a recently proposed approach for kinetics characterization in high-dimensional space,^25^ which we adapted here to biased MD simulations, to determine ligand unbinding pathways and to characterize both metastable and transition states that would otherwise be difficult to characterize experimentally.

The first step of this approach involved performing weighted principal component analysis (PCA) on the identified AMINO-based molecular features (9 for the morphine-bound MOPr system and 20 for the buprenorphine-MOPr system), as well as the reaction coordinate describing the drug’s hydration state, and the distance from D147^3.32^ used to define the unbinding events. Two clustering steps followed: the first one carried out solely for the purpose of determining the number of clusters and the second one using that cluster number to obtain clustered data for use in subsequent steps (see Ref. 25 for more information). Specifically, the data were projected onto the first two principal components and k-means clustering together with the Calinski–Harabasz test were used to determine the number of clusters that are appropriate for the system. The data were then projected onto the first three principal components and clustered by fitting it to a Gaussian Mixed Model (GMM) representation with the selected number of centers from the previous step. With these clustered data, we then built a higher-dimensional FES by fitting a GMM in *N*-dimensions, where *N* was taken to be 10 for both morphine and buprenorphine. Specifically, this was accomplished by calculating—with the previously clustered data—the mean, variance, and mixture weights using the *N* first principal components and accounting for the time-dependent weights (or bias) of each calculated feature.

To infer the transition paths and characterize the transition states, we used the nudged elastic band (NEB) algorithm on the *N*-dimensional GMM representation of the FES. The algorithm allows one to calculate the minimum-energy path (MEP) between adjacent cluster centers/minima corresponding to metastable states, as well as transition states corresponding to saddle points along the MEPs.^42^

In order to determine the rate-limiting steps of morphine and buprenorphine dissociation from the MOPr, and the corresponding transition states, we used the approach originally proposed by Pearce *et al.*^25^ In this approach, relative mean first passage times between states are obtained from a transition matrix whose entries are either given by Kramer’s rate constant if two minima are connected or zero otherwise. Note that, unless estimates of the effective friction coefficients are available, the strategy only predicts relative first passage times. The transition between adjacent FES minima with the largest mean first passage time represents the rate-limiting transition state.

## III. SELECTION OF MOLECULAR FEATURES AND REACTION COORDINATES TO PROPERLY STUDY LIGAND DISSOCIATION FROM MOPr

Using data from 20-ns unbiased simulations of either the morphine-MOPr or buprenorphine-MOPr complex, AMINO identified a reduced set of molecular features to be used as the input in RAVE to learn a reaction coordinate. In the case of morphine, these correspond to the following nine representative distances between morphine’s COM and mouse MOPr residues: V66^1.30^, L88^1.52^, D164^3.49^, F204^4.62^, D216^ECL2^, L254^5.60^, N274^6.29^, I302^6.57^, and V316^7.33×32^. For buprenorphine, the following 20 distances between the ligand’s COM and residues V66^1.30^, V80^1.44^, V92^1.56^, Q124^2.60^, M130^2.66^, I144^3.29^, D164^3.49^, I186^4.44^, Q212^ECL2^, D216^ECL2^, T220^ECL2^, W226^5.32×33^, L254^5.60^, V284^6.39^, I302^6.57^, E310^ECL3^, W318^7.35×34^, I322^7.39×38^, N332^7.49^, and I352^8.59×59^ were selected as representative molecular features of the system under study. Figure 2 of the supplementary material, generated by the GPCRdb website,^43^ shows the location of these residues on the receptor for both the simulated morphine- and buprenorphine-bound MOPr systems.

The aforementioned molecular features were used in combination with the unbiased trajectory data to train the RAVE NN, and the resulting reaction coordinate was used in an initial metadynamics simulation. The data from this metadynamics simulation were then used to train the RAVE NN again to obtain a better proxy of the reaction coordinate, which we then used in metadynamics production runs to achieve a more efficient and accurate calculation of the system’s kinetics. In the case of morphine, the reaction coordinate used in the infrequent metadynamics production runs was the following linear combination of the nine identified molecular features:

$$\begin{array}{ccc} & & 0.5d_{V66}-0.1d_{L88}+0.3d_{D164}+0.2d_{F204}-0.1d_{D216}\\ & & +\;0.3d_{L254}-0.3d_{N274}-0.1d_{I302}+0.7d_{V316}. \end{array}$$

For buprenorphine, the reaction coordinate learnt using the NN-based protocol was the following linear combination of the 20 identified molecular features:

$$\begin{array}{ccc} & & 0d_{V66}+0d_{V80}-0.1d_{V92}-0.3d_{Q124}+0d_{M130}+0.2d_{I144}\\ & & +\;0d_{D164}-0.1d_{I186}+0d_{Q212}-0.4d_{D216}-0.4d_{T220}\\ & & -\;0.6d_{W226}+0d_{L254}+0d_{V284}-0.2d_{\text{I}302}+0.2d_{\text{E}310}\\ & & +\;0d_{\text{W}318}+0.1d_{\text{I}322}+0d_{N332}-0.2d_{I352}. \end{array}$$

Interestingly, most of the coefficients are zero and, notwithstanding the larger number of AMINO features, the reaction coordinate for buprenorphine depends on a similar number of molecular features as the one for morphine (11 as compared to 9).

## IV. CALCULATED DISSOCIATION RATE CONSTANTS OF MORPHINE AND BUPRENORPHINE

**FIG. 2.**
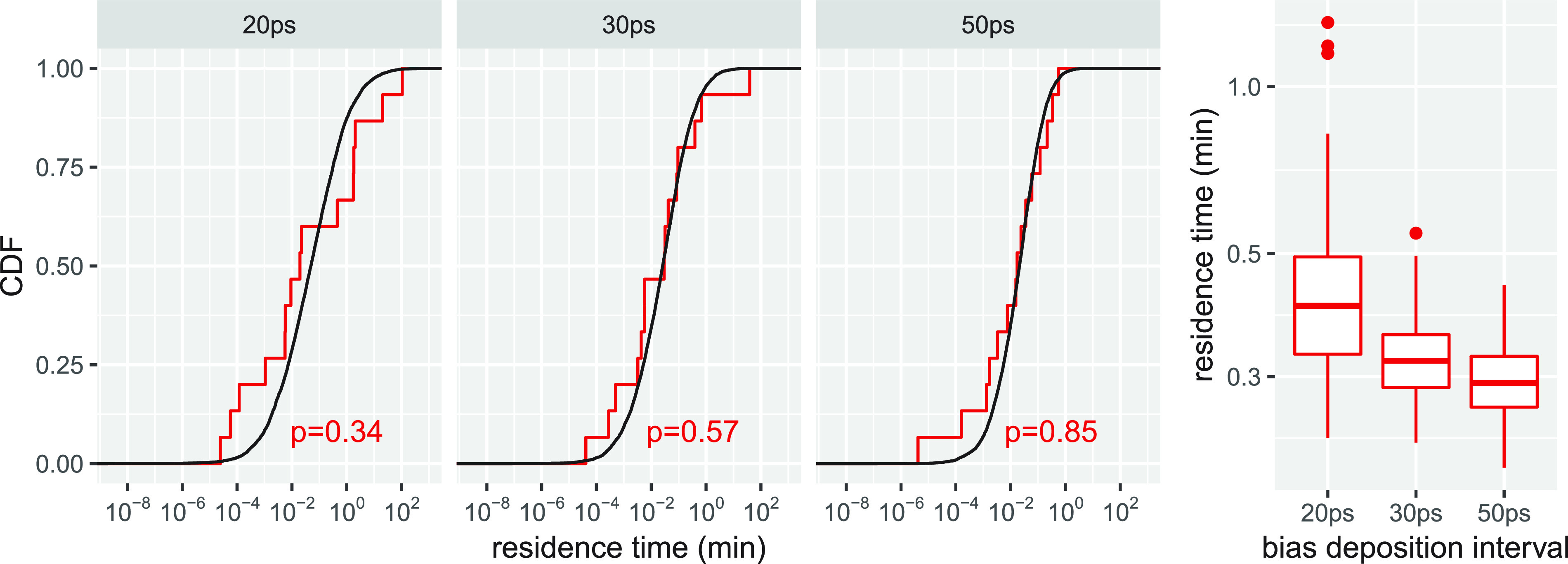
Comparison between the empirical cumulative distribution function (CDF) of morphine residence times derived from infrequent metadynamics simulations and the CDF of residence times derived from a theoretical model. [(a)–(c)] Results from infrequent metadynamics simulation runs with bias deposition intervals of 20 ps, 30 ps, and 50 ps respectively. The p-values for each fit derived from the KS test are also shown. (d) Range of morphine residence times, *τ*, calculated from the same simulations. Residence times are in unit of minutes, while bias deposition intervals are in picoseconds.

Bias deposition intervals of 20 ps, 30 ps, and 50 ps were used in separate infrequent metadynamics production runs to evaluate the speed-accuracy trade-off in simulating morphine dissociation from the MOPr as a test case. Our goal was twofold: (1) to assess how the bias deposition interval affected the confidence in the theoretical model we used for *τ* as judged by the fit of its cumulative distribution function (CDF) relative to the empirical CDF and (2) to verify the convergence of the predicted residence time in simulations using increasing bias deposition intervals. To quantify the fit between the two CDFs, we used the p-values derived from the KS test, which corresponded to 0.34, 0.57, and 0.85 for simulations of morphine-MOPr using bias deposition intervals of 20 ps, 30 ps, and 50 ps, respectively (Fig. 2). These simulations required increasing simulation times of ∼3 *µ*s, ∼4 *µ*s, and ∼6 *µ*s, representing an ∼7 order of magnitude speed-up relative to what would be required of unbiased MD simulations of morphine dissociation from the MOPr to reproduce experimental results (e.g., see Ref. 24).

The calculated residence time *τ* from these simulations was inverted for a direct comparison with *k*_off_ estimates from experiments. The calculated *k*_off_ values from simulations using bias deposition intervals of 20 ps, 30 ps, and 50 ps are 2.49 (1.95, 2.94) min^−1^, 3.19 (2.76, 3.61) min^−1^, and 3.44 (3.16, 3.78) min^−1^, respectively (Fig. 2). It is clear from Fig. 2 that the *k*_off_ values derived from simulations converge as a function of the bias deposition interval, with values derived from simulations with bias deposition intervals of 30 ps and 50 ps only marginally different. Importantly, all calculated *k*_off_ values for morphine compare well with the recently determined experimental value of 1.388 ± 0.1 min^−1^ from two-ligand competition experiments.^24^

**FIG. 3.**
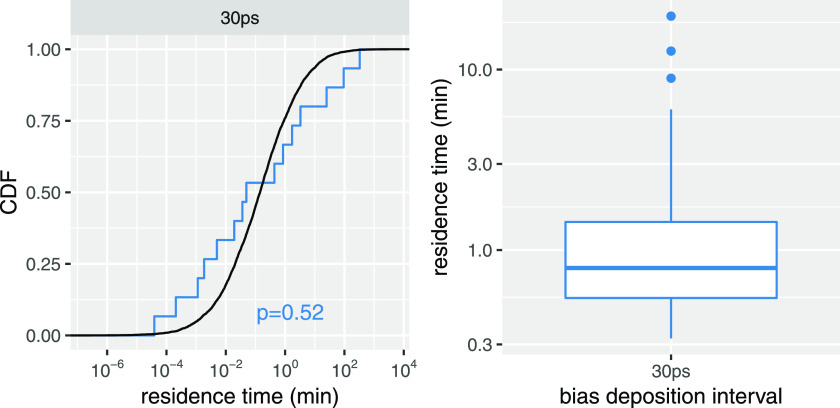
Comparison between the empirical cumulative distribution function (CDF) of buprenorphine residence times derived from infrequent metadynamics simulations and the CDF of residence times derived from a theoretical model. (a) Results from infrequent metadynamics simulation runs with a bias deposition interval of 30 ps. The p-value for the fit derived from the KS test is also shown. (b) Range of buprenorphine residence times, *τ*, calculated from the same simulations. Residence times are in unit of minutes, while bias deposition intervals are in picoseconds.

Since morphine-MOPr simulations using a bias deposition interval of 30 ps led to converged k_off_ values (Fig. 2), as well as a similar description of the drug’s dissociation process (see details in Sec. V), but at a much reduced computational cost (only 50% of the computational resources necessary to run simulations with a 50-ps bias deposition interval), we concluded that a bias deposition interval of 30 ps provides a good speed-accuracy trade-off to study the dissociation of morphinans from the MOPr. Thus, we only used a bias deposition interval of 30 ps to run the infrequent metadynamics simulations of the buprenorphine-MOPr complex. Similar to morphine, we ran 15 independent simulations, which required a total of ∼19 *µ*s. Figure 3 shows the fit of the theoretical CDF to the empirical CDF calculated using data from these simulations. Notably, the calculated p-value is similar to that obtained for morphine from simulations with a bias deposition interval of 30 ps. Also reported in Fig. 3 is the calculated *k*_off_ value for buprenorphine derived from these simulations, which is 1.27 (0.84 and 1.62) min^−1^. While this calculated value is approximately one order of magnitude different from the recently published experimental result of 0.106 ± 0.02 min^−1^,^24^ we note that (a) it is reasonable considering the intrinsic limitations of the force-field model, and (b) it shows the right trend by predicting a slower residence time for buprenorphine compared to morphine, in line with experiments.^24^

## V. COMPARISON BETWEEN MORPHINE AND BUPRENORPHINE DISSOCIATION MECHANISMS FROM MOPr

**FIG. 4.**
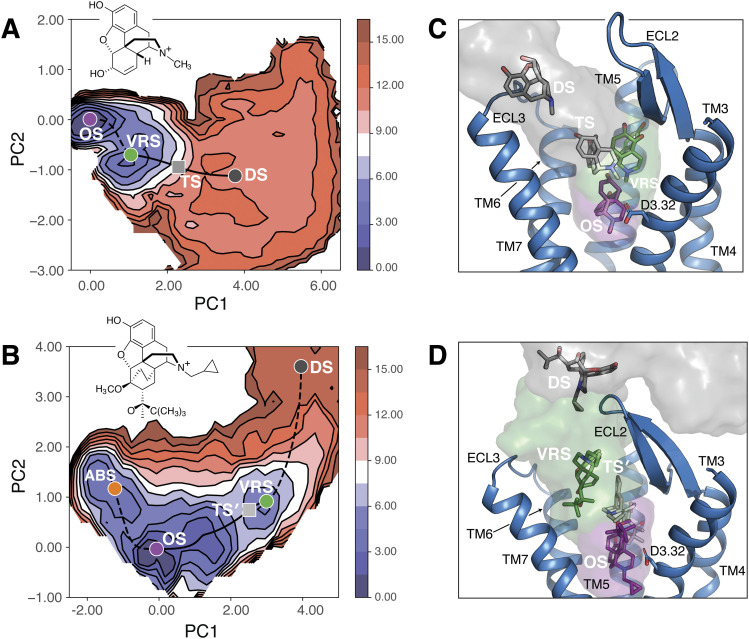
Energetic and structural descriptions of the morphine-MOPr and buprenorphine-MOPr dissociation processes. FES of (a) morphine and (b) buprenorphine projected onto the first two principal components from PCA of the combined AMINO-based molecular features, the reaction coordinate describing the drug’s hydration state, and the distance from D147^3.32^ used to define an unbinding event. The MEPs predicted by the NEB method are plotted onto these FES as black dashed lines connecting cluster centers/minima (colored dots), unless they refer to the rate-limiting steps in which case they are shown as solid black lines. The transition states along these paths (TS and TS′ for morphine-MOPr and buprenorphine-MOPr, respectively) are shown as gray squares. [(c) and (d)] Representative conformations for each minimum, including the orthosteric ground state (OS; purple), the vestibule region state (VRS; green), and the solvent-exposed/dissociated states (DS; dark gray) as well as for the rate-limiting transition states (TS and TS′ ; light gray). MOPr TM helices are shown in cartoon representations with TM1 and TM2 helices not displayed for clarity.

We compared the molecular mechanisms underlying morphine and buprenorphine dissociation from the MOPr, using data from infrequent metadynamics simulations of each system with a bias deposition interval of 30 ps. Figures 4(a) and 4(b) show the FES describing morphine-MOPr and buprenorphine-MOPr dissociation processes, respectively, projected onto the first two principal components from PCA of the combined AMINO-based molecular features, the reaction coordinate describing the drug’s hydration state, and the distance from D147^3.32^ used to define an unbinding event. MEPs predicted for each system by the NEB method are plotted onto the FES plots as dashed lines connecting cluster centers/minima (colored dots), unless they refer to the rate-limiting steps in which case they are indicated by solid lines. While color dots refer to the lowest energy (ground) state (herein called “orthosteric state;” OS) or metastable states (“alternative bound state,” “vestibule region state,” and “dissociation state;” ABS, VRS, and DS, respectively), the gray squares refer to rate-limiting transition states (TS and TS′ for morphine-MOPr and buprenorphine-MOPr, respectively). We note that the estimation of the relative MFP times and the extraction of the representative states were performed in the high-dimensional space, and the relative location of the minima and transitions shown on the free-energy projection onto the first two PCA components in Fig. 4 is intended for visualization purpose only. Further validation of the transition states was provided by committor analysis. Using the morphine-MOPr system as an example, we extracted ten transition state configurations from each of the three representative simulations of morphine dissociation from the MOPr carried out with a 30-ps bias deposition interval and ran 20 parallel, independent simulations for each of these transition state configurations, for a total of 600 simulations. From these calculations, we obtained a committor probability of 0.52 in line with the expectation that a transition state would have equal probability of falling in either of the adjacent wells. Structural representatives of each identified metastable and transition state are shown in Figs. 4(c) and 4(d) for the morphine-MOPr and buprenorphine-MOPr systems, respectively, with the exception of buprenorphine’s ABS, which is shown in Fig. 3 of the supplementary material.

As can be seen in Fig. 4(a), the dissociation of morphine from the MOPr can be described as a two-step transition, starting from what we call the ground “orthosteric state” at the crystallographically identified orthosteric site for morphinans [purple in Figs. 4(a) and 4(c)]. This state transitions to an intermediate state that we call the “vestibule region state” [green in Figs. 4(a) and 4(c)], prior to reaching a much more “solvent-exposed state” [gray in Figs. 4(a) and 4(c)], which includes poses of fully dissociated morphine. The NEB method predicts the barrier heights for the transitions involving the vestibule region state of morphine to be significantly different (1.6 kcal/mol vs 19.0 kcal/mol), with the much lower barrier height referring to the molecule transitioning back to the orthosteric state as opposed to transitioning to the solvent-exposed state. In addition, the barrier height for the initial transition of morphine from the orthosteric state to the vestibule region state is 7.5 kcal/mol, which is also significantly smaller than the barrier height for the final transition to the solvent-exposed state (19.0 kcal/mol), suggesting that morphine transitions back and forth between the orthosteric state and the vestibule region state prior to moving to a final solvent-exposed state. This suggests that it takes longer for morphine to transition from the vestibule region state to the solvent-exposed and dissociated state, and therefore, the rate-limiting transition state for morphine corresponds to the final dissociation step. This is confirmed by the matrix of first passage times shown in Table S2, which predicts that the final dissociation step is indeed the rate-limiting step. Notably, similar results were obtained from the infrequent metadynamics simulations of the morphine-MOPr using the longer bias deposition interval of 50 ps (see Fig. 4 of the supplementary material).

**FIG. 5.**
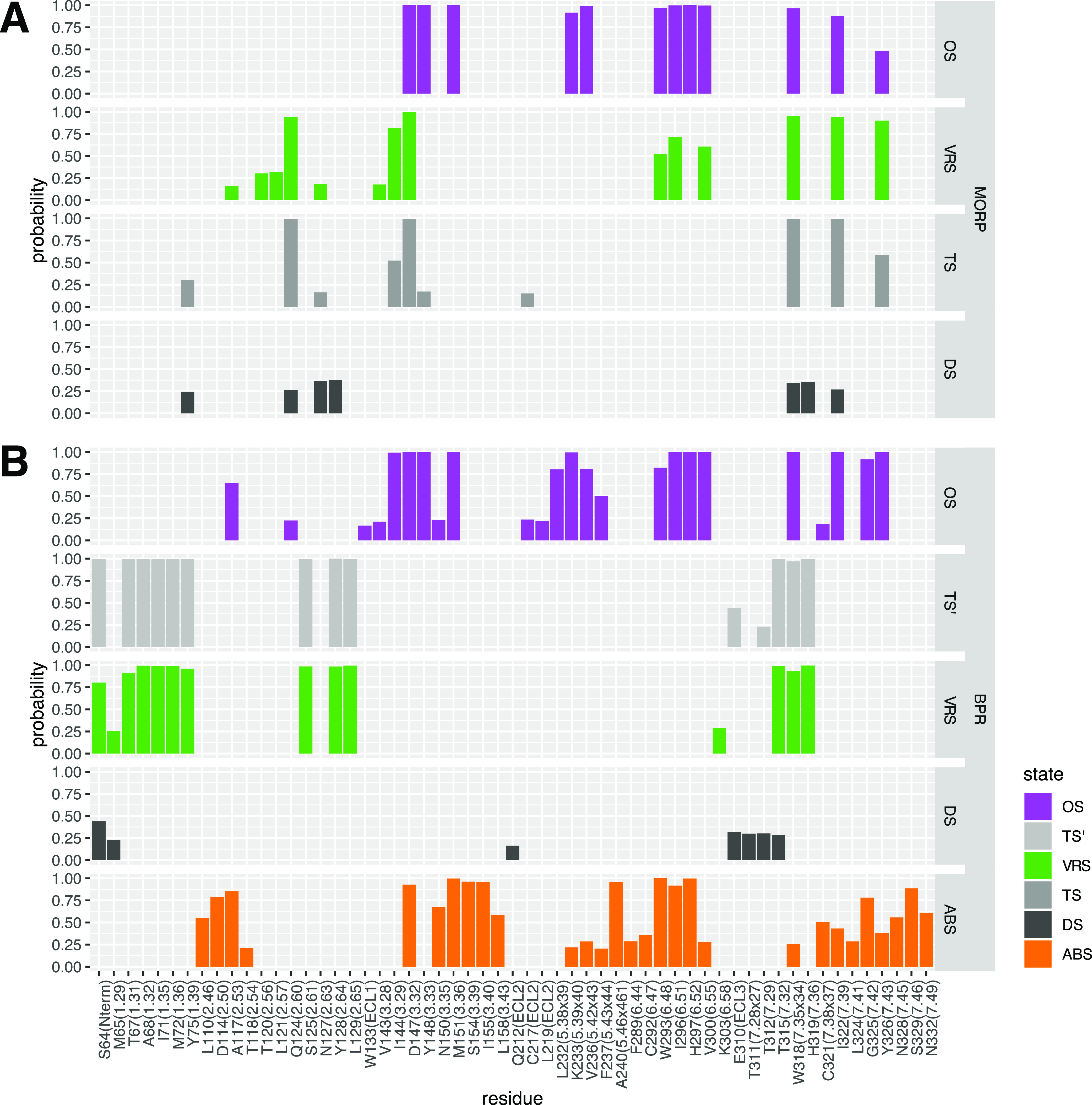
Fractions of contacts formed by morphine or buprenorphine during dissociation from the MOPr as simulated by infrequent metadynamics with a bias deposition interval of 30 ps. (a) Morphine-MOPr and (b) buprenorphine-MOPr contact fractions in the metastable states and rate-limiting transition states identified along the drugs’ dissociation paths. The contacts were defined as the probability that the minimum distance between heavy atoms of the ligand and each MOPr residue is below 5 Å. Only contacts with a probability larger than 15% are displayed.

The first notable difference between morphine and buprenorphine dissociation paths from the MOPr is the presence of an alternative binding state at the orthosteric site, whose representative structure is shown in detail in Fig. 3 of the supplementary material to avoid overcrowding of Fig. 4(d). Another notable difference is that the vestibule region state of buprenorphine extends much further into the extracellular region of the MOPr than morphine, overlapping with representatives of the final, solvent-exposed state of morphine. Furthermore, the final, solvent-exposed state of buprenorphine includes representative structures interacting with residues of the MOPr extracellular loop (ECL) region, particularly ECL2, that were much less likely to be involved in interaction with morphine, reflecting morphine’s tendency to reach a freely diffusing state much more quickly compared to buprenorphine. The barrier heights predicted by the NEB method for the transition between the orthosteric state and the alternative bound state of buprenorphine, in both the forward and reverse directions, are significantly lower (9.9 kcal/mol and 1.6 kcal/mol, respectively) than that for transitioning from the orthosteric state to the vestibule region state (28 kcal/mol). Only the orthosteric state of buprenorphine can transition to the vestibule region state with the alternative bound state able to only transition back to the orthosteric state. Once buprenorphine reaches the vestibule region state, it needs to overcome similar barrier heights to proceed in either direction. Thus, the longest transition time for buprenorphine corresponds to the transition from the orthosteric state to the vestibule region state, while the transition time for buprenorphine to move from the vestibule region state to a fully dissociated state, or back to the orthosteric state, is four and three orders of magnitude faster, respectively. This observation led us to conclude that the rate-limiting transition state in buprenorphine dissociation corresponds to the second step of the process, when the molecule transitions from the orthosteric to vestibule region state, as opposed to the last step for morphine (i.e., from the vestibule region state to the solvent-exposed and fully dissociated states), notwithstanding some overlap between the vestibule region state of buprenorphine and the final state of morphine. Notably, hydration appears to have an important role in the slower dissociation rate of buprenorphine compared to morphine as judged by plots of the potential of mean force as a function of the ligand hydration state (see Fig. 5 of the supplementary material). The crucial role of buprenorphine lipophilicity on the kinetic properties of this ligand has been hypothesized in the past,^44,45^ and our simulations provide insight into the underlying molecular mechanism. Specifically, a clear barrier of ∼2 kcal/mol is visible in the transition between the orthosteric state and the vestibule region state in the simulated buprenorphine-MOPr system, whereas no clear barrier is observed in the morphine’s rate-limiting transition between the vestibule region state and the ligand’s dissociated state [compare Figs. 5(a) and 5(b) of the supplementary material]. Notably, the average ligand hydration of 2.2 for buprenorphine at the rate-limiting transition state [see Fig. 5(b) of the supplementary material] is significantly lower than morphine’s ligand hydration [3.6; see Fig. 5(a) of the supplementary material]. This observation suggests that the longer residence time of buprenorphine in the MOPr may depend on the various hydrophobic moieties of buprenorphine, which contribute to its decreased hydration and consequently hindered transition of the ligand from the orthosteric to vestibule region.

## VI. STRUCTURAL FEATURES OF MORPHINE-MOPr AND BUPRENORPHINE-MOPr STATES ALONG DRUG DISSOCIATION PATHS

Figure 5 shows the drug-MOPr contact fractions (defined as the probability that the minimum distance between heavy atoms of the drug and each MOPr residue is below 5 Å) calculated after data reweighting for each state identified along the drug’s dissociation paths by infrequent metadynamics runs with a bias deposition interval of 30 ps. As shown in Fig. 5(a), the lowest energy state of morphine (i.e., the orthosteric state) is characterized by the largest number of high contact fractions with residues located in TM helices 3, 5, 6, and 7. In addition to the well-known contact with D147^3.32^, this orthosteric bound state of morphine (purple color in Figs. 4 and 5) is characterized by a series of high-contact fractions (>75%) with a number of residues within the crystallographic binding pocket of morphinans, namely, Y148^3.33^, M151^3.36^, K233^5.39×40^, V236^5.42×43^, W293^6.48^, I296^6.51^, H297^6.52^, V300^6.55^, W318^7.35×34^, and I322^7.39×38^.

Similar to the orthosteric bound state, the identified vestibule region state of morphine (green in Figs. 4 and 5) is characterized by high fractions of contacts (>75%) formed by the drug with D147^3.32^, W318^7.35×34^, I322^7.39×38^, and Y326^7.43×42^. However, interactions of morphine with residues Y148^3.33^, M151^3.36^, K233^5.39×40^, V236^5.42×43^, and H297^6.52^ are lost completely in this state, while interactions with W293^6.48^, I296^6.51^, and V300^6.55^ are still formed, albeit with reduced fractions. Instead, in this vestibule region state, morphine appears to prefer interactions with TM2 residues, and in particular with Q124^2.60^ (contact fraction >75%). The solvent-exposed/dissociated state of morphine (dark gray in Figs. 4 and 5) forms only weak interactions, with the largest contact fractions (∼30%–40%) calculated for residues Q124^2.60^, N127^2.63^, Y128^2.64^, W318^7.35^, and H319^7.36^.

The rate-limiting transition state of morphine (TS in Figs. 4 and 5) corresponds to the transition from the vestibule region state to the solvent-exposed and dissociated states. The drug in TS forms the highest fractions of contacts (>75%) with Q124^2.60^, D147^3.32^, W318^7.35×34^, and I322^7.39×38^ and interacts to a smaller degree (contact fraction ∼30%–40%) with I144^3.29^ and Y326^7.43×42^. The main difference between the rate-limiting TS and the vestibule region state is the loss of interactions with residues W293^6.48^, I296^6.51^, and V300^6.55^ by the drug in the TS. Notably, contact fractions characterizing the morphine states identified along the drug’s dissociation path by infrequent metadynamics simulation runs with a bias deposition interval of 50 ps show similar trends to those described above (see Fig. 6 of the supplementary material).

The fractions of contacts formed by buprenorphine with MOPr residues during its dissociation simulated by infrequent metadynamics runs with a bias deposition interval of 30 ps are shown in Fig. 5(b). As can be seen in this figure, many of the highest fractions of contacts (>75%) formed by buprenorphine with the MOPr in its lowest energy orthosteric bound state (OS), purple in Figs. 4(d) and 5(b), coincide with those formed by morphine in its own orthosteric state. Specifically, these contacts are formed with residues D147^3.32^, Y148^3.33^, M151^3.36^, K233^5.39×40^, V236^5.42×43^, W293^6.48^, I296^6.51^, H297^6.52^, V300^6.55^, W318^7.35×34^, and I322^7.39×38^. However, due to its larger size, buprenorphine is found to establish additional high contact fractions (>75%) with L232^5.38×39^ and G325^7.42^ as well as to enhance its interaction with Y326^7.43×42^ compared to morphine. In the alternative bound state in which buprenorphine—but not morphine—is found to populate, the drug continues to form high fractions of contact (>75%) with D147^3.32^ but also with other residues involved in the orthosteric bound state of buprenorphine, namely, M151^3.36^, W293^6.48^, I296^6.51^, and H297^6.52^. However, in this alternative bound state of buprenorphine, the interactions with residues K233^5.39×40^, V236^5.42×43^, V300^6.55^, W318^7.35×34^, I322^7.39×38^, and Y326^7.43×42^ are significantly reduced, while disappearing completely for residues I144^3.29^, Y148^3.33^, and L232^5.38×39^. New interactions that are formed with the highest probability (>75%) in the alternative bound state are D114^2.50^, S154^3.39^, I155^3.40^, A240^5.46×461^, and S329^7.46^.

Breaking of the interaction between buprenorphine and D147^3.32^ is one of the main events marking buprenorphine’s transition from its orthosteric site to the vestibule region state. This is a significant difference between the vestibule region states of buprenorphine and morphine, with morphine maintaining its interaction with D147^3.32^ in this metastable state. In the vestibule region state, buprenorphine interacts with a completely different set of residues compared to its orthosteric and alternative bound states with the only exception being W318^7.35×34^. Specifically, the drug forms the highest fractions of contacts with residues S64 (N-term), T67^1.31^, A68^1.32^, I71^1.35^, M72^1.36^, Y75^1.39^, S125^2.61^, Y128^2.64^, L129^2.65^, T315^7.32×31^, W318^7.35×34^, and H319^7.36×35^. The state containing the most solvent-exposed and dissociated conformations of buprenorphine [dark gray in Figs. 4(b) and 5(b)] mainly forms low-frequency interactions with the N-terminus, TM1, TM7, and ECL3. Among them, the buprenorphine’s highest contact fractions (∼30%–40%) in this state involve S64 (N-term) and the E310 residue in ECL3.

The representative structure of the rate-limiting transition state of buprenorphine (TS′) retains, with high contact fractions, most of the drug interactions seen in the vestibule region state. Notable differences between the rate-limiting transition state and the vestibule region states of buprenorphine are (a) the complete loss of interaction with residues M65^1.29^ and K303^6.58^ and (b) the formation of contacts with relatively low probability (∼45%) involving the ECL3 residue E310, which is also observed in the solvent-exposed/dissociated state. Also interesting in light of the observed dissociation rate differences between morphine and buprenorphine is that the rate-limiting transition state identified along the dissociation path of buprenorphine from the MOPr has a broken salt bridge with D147^3.32^, whereas this interaction is still present in the rate-limiting transition state of morphine, contributing to its stabilization and consequent shorter residence time of the drug.

## VII. SUMMARY AND CONCLUSIONS

We have investigated in this work the efficiency and accuracy of a computational strategy that combines machine learning and infrequent metadynamics to simulate drug dissociation from GPCRs, a process that, depending on the ligand, may require minutes or even hours in real life. Specifically, we investigated the binding of two representative morphinan drugs with different MOPr residence times by using two machine learning algorithms to learn an appropriate drug dissociation reaction coordinate to carry out infrequent metadynamics simulations aimed at providing both kinetic rates and mechanistic information. The strategy proved effective in (a) achieving from microsecond-timescale simulations the dissociation of ligands that typically unbind in 1 min–10 min, which corresponds to ∼7 orders of magnitude speed-up, (b) predicting *k*_off_ values for morphine and buprenorphine that are in agreement with the experimental observation that the bound state of buprenorphine is more long-lived than morphine, and (c) providing mechanistic hypotheses of dissociation kinetics that, once validated experimentally, may be useful for designing improved therapeutics.

It must be noted that the expectation from these or other MD simulations was not to obtain *k*_off_ values that were identical to experiments. Various factors, including—but not limited to—force-field approximations^15^ and differences in the lipid environment between a cell membrane and a simplified lipid bilayer used in simulations, make these quantities difficult or impossible to compare in absolute terms. In addition to the recognized importance of incorporating ligand solvation for both binding pose predictions^46,47^ and binding free energies,^48^ we have found (similar to the conclusions of Ref. 16) that ligand solvation is an important coordinate to be considered for accurate rate estimates. Our initial tests ignoring ligand solvation led to unreasonable *k*_off_ values (i.e., slower by ∼7 orders of magnitude) for the systems under study (data not shown). Although we could have added this coordinate to the list of molecular features used as the input for NN and incorporated into the RAVE RC, we chose to use ligand solvation as a second biasing CV to preserve the RAVE’s currently validated NN architecture and number of bottleneck neurons.

We were pleased to see that our strategy is capable of predicting a *k*_off_ ratio for morphine and buprenorphine that is only one order of magnitude from experiments. These results attest to the effectiveness of the RCs used to simulate the dissociation of rigid chemical scaffolds such as morphinans from their target GPCR despite the fact that the molecular features used as the input for AMINO were based on the ligand-bound states. This choice, therefore, hinders the application of the current protocol to cases in which there is no prior knowledge of the binding site or where the dissociation process involves major conformational rearrangements of either the ligand or the protein. For instance, we anticipate that simulating the unbinding of a larger and more flexible ligand might require taking into account the conformational plasticity of both the ligand and the protein. In this case, one might derive more appropriate molecular features by studying the dynamics of the system. In situations where the ligand binding site is known experimentally, docking strategies^49,50^ may be sufficient to generate starting poses for simulations (biased or not) aimed at producing a complementary set of molecular features related to ligand dissociation. Should the binding site be unknown, using enhanced sampling approaches may offer a better strategy to explore conformational flexibility of both the protein and the ligand, as well as binding sites and pathways. Notably, although a Poissonian behavior does not guarantee that the RC used to describe ligand unbinding is correct, a poor choice of CVs results in deviations from homogeneous Poisson behavior of the distribution of dissociation times.^41^ These deviations can be detected by poor CDF fits notwithstanding increasingly infrequent deposition rates. Although the protocol can in principle be extended to more complex cases, it should also be noted that simulating, for instance, a larger and more flexible ligand might require longer bias deposition intervals to achieve a good fit between the empirical and theoretical CDFs as well as longer infrequent metadynamics simulation times to converge the residence times. We note that the simulations of buprenorphine, which is only slightly larger than morphine, already took ∼19 *µ*s to achieve simulation convergence.

Admittedly, the use of linear combinations of input molecular features to derive RCs such as in RAVE shares similarities with other RC building methods. For instance, the work of McCarty and Parrinello^51^ also uses linear combinations of input molecular features and well-tempered metadynamics to derive RCs and has subsequently been applied to estimate residence times.^52^ To estimate the coefficients describing the linear mapping, however, McCarty and Parrinello solved a generalized eigenvalue problem, while RAVE derives coefficients from the data by training a NN. Similarly, some published time-independent component analysis (tICA)-based approaches have also made use of both unbiased and biased data to obtain RCs from linear combinations of molecular features.^53–55^

An important outcome of MD-based computational strategies such as the one reported herein is the mechanistic information one can derive for the process of interest, in this case, the dissociation of morphinan ligands from the MOPr. Using this mechanistic information, alongside an application of the NEB method to the metastable structures obtained from GMM clustering, it is possible to determine the rate-limiting transition structures that govern the overall dissociation kinetics of the drug-target complex. In the case of morphine, the rate-limiting step of its dissociation from the MOPr corresponds to the transition from the vestibule region state to the solvent-exposed and dissociated state. Several morphine-MOPr contacts, specifically, those involving residues I144^3.29^, W293^6.48^, I296^6.51^, V300^6.55^, and Y326^7.43×42^, are considerably reduced or lost completely in the transition from the vestibule region state to the rate-limiting transition state. Among the interactions that stabilize the rate-limiting transition state of morphine dissociation from the MOPr, those involving the following residues have high probability: Q124^2.60^, D147^3.32^, W318^7.35×34^, and I322^7.39×38^. Destabilizing any of these interactions would increase the energy barrier for transitioning from the vestibule region state to the solvent-exposed and dissociated state, resulting in a longer residence time for this drug.

Unlike morphine, the rate-limiting transition state governing the overall kinetics of buprenorphine dissociation from the MOPr is predicted to correspond to the transition from the orthosteric bound state to the vestibule region state. There are two weak interactions formed by buprenorphine in the rate-limiting transition state, which are neither present in the vestibule region state nor in the orthosteric bound states. These interactions are formed with residues in close proximity to one another in the receptor, namely, ECL3 E310 and T312^7.29×28^ (43% and 23%, respectively). On the other hand, a number of contacts are newly formed in the buprenorphine’s rate-limiting transition state. Notably, the majority of these contacts are with residues in TM1 and TM2 (T67^1.31^, A68^1.32^, I71^1.35^, M72^1.36^, Y75^1.39^, S125^2.61^, Y128^2.64^, and L129^2.65^) but also involve a couple of residues in TM7 (T315^7.32×31^ and H319^7.36^). Destabilizing any of the aforementioned interactions might further delay the transition of buprenorphine from the bound state to the vestibule region state.

One way to achieve destabilization of rate-limiting transition states and possibly increase the drug residence time is through chemical modification of the drug. For instance, the orientation of morphine in the rate-limiting transition state is such that the drug interacts with W318^7.35×34^ via (i) a π–π interaction between its benzene ring incorporating a phenolic hydroxyl group and the residue aromatic ring, (ii) a hydrogen bond between its hydroxyl group at the 6-position and the residue indole nitrogen, and (iii) a hydrophobic interaction between the ring incorporating the hydroxyl group at the 6-position and I322^7.39×38^. The last two of these interactions are also formed in the vestibule region state, but they are not as prevalent as in the rate-limiting transition state. Thus, chemical modifications of morphine that would destabilize these specific interactions are predicted to have the largest impact on the energy barrier height and to lead to longer RT of the morphine scaffold. For instance, replacing the aforementioned hydroxyl group at the 6-position with a longer hydroxyl chain or an alkyl group that reduces the polarity while adding steric hindrance might offer a strategy for increasing the residence time of morphine. Experimental testing of these types of hypotheses would be important for a kinetic-oriented drug design.

## SUPPLEMENTARY MATERIAL

See the supplementary material for Tables S1 and S2 and Figs. 1–6.

## DATA AVAILABILITY

The data that support the findings of this study are available from the corresponding author upon reasonable request.
